# Associative learning by *C. elegans* is variable when butanone is paired with starvation

**DOI:** 10.17912/micropub.biology.001568

**Published:** 2025-05-13

**Authors:** Samiha Tasnim, Amber Liu, Antony Jose

**Affiliations:** 1 Cell Biology and Molecular Genetics, University of Maryland, College Park, College Park, Maryland, United States

## Abstract

The nematode *C. elegans* has been reported to show a reduction in its preference for the odorant butanone after prior exposure to butanone coupled with starvation. Here we report unexplained variability in such associative learning. Pre-exposure of unfed worms to butanone resulted in different responses during different trials of subsequent chemotaxis assays – from strong avoidance to enhanced attraction. Given this variation in associative learning despite the artificially controlled lab setting, we speculate that in dynamic natural environments such learning might be rare and highlight the challenge in discovering evolutionarily selected mechanisms that could underlie learning in the wild.

**Figure 1. Learned association of butanone with starvation by wild-type worms is variable:**
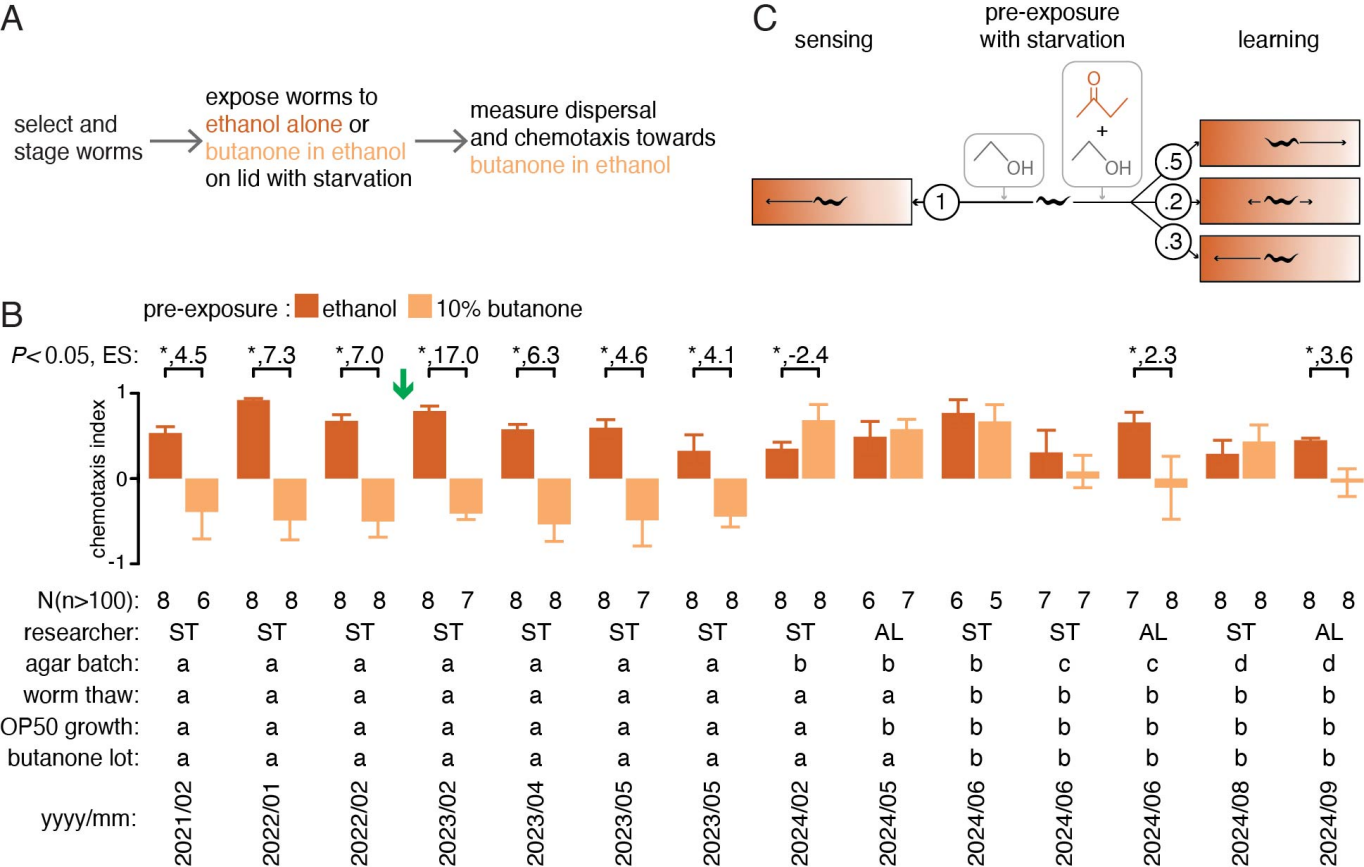
*(A)* Schematic summary of assay for associative learning, if any, when butanone is paired with starvation (adapted from Tasnim et al., 2025). *(B)* In the absence of food, worms pre-exposed to the vehicle ethanol consistently showed attraction to butanone , however worms pre-exposed to butanone showed variable responses (including aversion, reduced attraction, and increased attraction) when different cohorts of worms were tested over a period of ~2.5 years. Significant differences ( *P* < 0.05 using Student's t-test, *), effect sizes for significant differences (Cohen's *d,* ES), 95% confidence intervals (error bars), populations tested (N), numbers of worms in each population required for interpretation (n), researcher performing the experiment (ST or AL), agar batch (a, b, c, or d), worm thaw (a or b), OP50 growth (a or b), and butanone lot (a or b) are shown. Green arrow indicates when the dispersal plates were added as an additional control. *(C)* Summary schematic of *C. elegans* responses to butanone . Numbers represent proportions of assays showing each type of response.

## Description

Despite their short lives and complex environments, *C. elegans* have been reported to be capable of associative learning (reviewed in Zhang et al., 2024). A ~1 hr pre-exposure to ‘attractive' odorants in the absence of food eliminates the attraction when tested using a subsequent assay and such learning, that presumably associates starvation with the odorant, was not observed if the pre-exposure was done in the presence of food (Nuttley et al., 2002). Incorporating the pre-exposure to butanone with starvation into a recently developed assay for chemotaxis (Fig. 1 *A* ; Tasnim et al., 2025) resulted in worms moving away from butanone – an altered response that was reproduced in 6 subsequent trials (Fig. 1 *B* ; effect sizes ranging from ~4.1 to ~17.0). This apparent associative learning was eliminated when the pre-exposure was performed in the presence of food (Supplemental Dataset 1; chemotaxis index (CI) after ethanol pre-exposure = 0.75 +/- 0.12 and CI after butanone pre-exposure = 0.8 +/- 0.18). However, in 7 subsequent trials without food the measured associative learning, if any, varied widely (effect sizes from -2.4, indicating increased attraction to butanone , to 3.6, indicating decreased attraction to butanone ). The previously detected learned butanone avoidance could not be reproduced despite varying agar (2 trials each using 2 new sources), researcher (3 trials by one and 4 by another), worms (5 trials using a 2 ^nd^ isolate), *E. coli* (6 trials using a 2 ^nd^ isolate), and butanone (5 trials using a 2 ^nd^ lot). In contrast, the initial response to odorant sensation remained reproducible ( Fig. 1B ) and initial responses to a different attractive odorant benzaldehyde and an aversive odorant nonanone were also reproducible (Tasnim et al., 2025). Artificially categorizing the responses measured in each trial (Fig. 1 *B* ) into trials detecting learned avoidance and trials with no detectable learning enables an evaluation of the pattern using the runs test (Swed and Eisenhart, 1943; Bujang and Sapri, 2018). This test does not support a significant deviation from random (r = 5 > L _c_ = 3; see Statistics in Materials and Methods).

Our measurements of behavior without immobilization reveal that unlike the reproducible response upon sensing an odorant (Tasnim et al., 2025), the observed response after associative learning is variable and not distinguishable from random (Fig. 1 *C* ), suggesting that reproducible learning, if any, requires artificial environments that are not easily controlled. Under this hypothesis, past reports of learning (reviewed in Zhang et al., 2024) likely include undeclared or cryptic experimental conditions (e.g., unknown aspects of agar (Bargmann, 2024)) that are needed for the reproducible detection of associative learning. The discovery of such conditions will support the wider study of the learning detected in the laboratory. Another possibility is that robust learning by *C. elegans* has been lost through growth in the laboratory (Frézal & Félix, 2015). Finally, in the dynamic natural environment of *C. elegans* where evolutionary forces sculpt molecular mechanisms, learning to associate an odorant with starvation could be rare.

## Methods

**Worm growth and selection**

The *C. elegans* wild type Bristol N2 was obtained from the Caenorhabditis Genetics Centre (University of Minnesota, Minneapolis, MN, USA). Worms were grown at 20ºC, and chemotaxis assays were performed at room temperature (~25ºC).

Growth, pre-exposure, and assay plates were prepared using appropriate volumes of Nematode Growth Medium (NGM): 4 ml per 35 mm plate, 20 ml per 100 mm plate and 8 ml per assay plate. NGM was prepared by combining 17 g bacteriological agar, 2.5 g bacto-peptone, and 3 g NaCl in 1 L water, autoclaving, cooling to 60ºC, then adding 1 ml each of cholesterol (5mg/ml in ethanol), 1 M CaCl _2_ and 1 M MgSO _4_ , and 25 ml of potassium phosphate buffer (pH 6.0) (see Table 1). The use of various sources of agar was inspired by anecdotal reports that behavior can vary between batches of agar (e.g., (Bargmann, 2024)).

Growth plates were seeded with overnight cultures of *E. coli* OP50 grown at 37ºC in Luria-Bertani (LB) broth (100 µl per 35 mm NGM plate and 500 µl per 100 mm NGM plate) and left at room temperature for ~48 hrs, by when a lawn forms. These seeded plates were stored for up to 4 weeks at 4ºC until use. Worm washes and transfers were done using M9 buffer prepared by adding 5 g NaCl, 11.32 g dibasic Na _2_ HPO _4_ *7H _2_ O and 3 g KH _2_ PO _4_ to 1 L water, autoclaving, and cooling to ~60ºC before adding 1 ml of 1 M MgSO _4_ . A worm bleaching solution (a stock mix of 45 ml 5 M NaOH, 15ml Clorox® bleach (8.25% NaOCl) and 90 ml water aliquots diluted to 50% before use) was used to dissolve worms while leaving the embryos protected by the eggshells. Subsequent hatching resulted in populations of L1-staged animals that were then moved to seeded plates and grown to adulthood before their behavior was assayed. Bleaching was performed only once per experimental replicate and bleached worms were not propagated; unbleached worms were separately maintained as the stock.

**Staging**

Plates were set up on day 1 by washing an unstarved but crowded 35 mm plate of worms with 1 ml M9 and transferring 10 µl of the suspension onto 12 OP50-seeded 35 mm plates. These plates were grown for 36-48 hrs at 20ºC until large numbers of gravid adults were observed, then worms were bleached by dropwise addition of 250-300 µl of worm bleaching solution, ensuring that all worms were covered using the least volume of bleaching solution to facilitate quick diffusion of bleach into the agar thereby minimizing stress on worms that hatch from the surviving eggs. Bleaching was chosen in contrast to precise staging by picking L4-staged larvae because of the need for hundreds of worms for each experiment and to ensure uniform treatment of all worms (e.g., differences in mechanical handling of worms could impact subsequent behavior). Bleached plates were kept at 20ºC for 24 hrs until the surviving eggs hatched, then L1 worms were pooled from 2 plates by washing with 500 µl M9 and transferred onto a seeded 100 mm plate. These worms were then allowed to grow for an additional 72 hrs (total 96 hrs post-bleach) to get a synchronized cohort of young adult worms which were then pre-exposed and tested for their response to volatile odorant(s). Six 100mm plates of young adult worms were sufficient to pre-expose with one odorant (or vehicle) and to measure both dispersal and chemotaxis in 8 arenas each.

**Pre-exposure**

M9 buffer (5-6 ml) was used to pool worms from six 100 mm plates into 1.5 ml microcentrifuge tubes and washed a total of 3 times by adding 1 ml of M9 and centrifuging at 11,000 rpm for 2 min. This use of centrifugation is different from the gravity-aided settling that was used in some assays for associative learning (Kaufmann et al., 2011) (we estimate gravity-aided settling until the solution above the worm ‘pellet' is clear to take >7 min, Movie S1). However, centrifugation was consistently used in all trials, including the 9 repeats that showed learning with an effect size >2 (Fig. 1 *B* ). At the end of the third wash, the supernatant was discarded, retaining 200 µl of liquid with worms and one such tube of worms was used per 100 mm plate during pre-exposure. A P1000 pipette was used to transfer worms onto 100 mm plates with (for one experiment) or without *E. coli* OP50 food, taking care not to transfer any bacterial pellets and pipetting from the top of the worm “pellet” so as not to transfer any worm carcasses from the bottom of the tube. Ethanol was used as the vehicle and 2- butanone as the test odorant. Both vehicle and odorant stocks were stored in airtight glass bottles protected from the light, while aliquots of each in 1.5 ml Eppendorf tubes were kept for no more than 4 weeks. Fresh odorant dilutions (10% 2- butanone ) were made on the day of the behavioral assay in 1.5 ml microcentrifuge tubes. 5 µl of either the vehicle or the diluted odorant was streaked onto the lid of the plate and the covered plate was then sealed with parafilm. Plates were kept undisturbed at room temperature for 1 hr, then pre-exposed worms were collected and washed three times using M9 buffer. At the end of the third wash the supernatant was discarded, retaining ~200 µl of liquid with worms.

**Chemotaxis and dispersal assays**

Arenas were set up as described (Fig. 1 *A* in Tasnim et al., 2025) and for each test (dispersal or chemotaxis), 2 sets of 4 rectangular arenas were used per pre-exposure treatment (N=8). A template was used to trace quadrants onto extra lids of plates and these lids were placed under each set of 4 arenas to aid transfer of worms to the origins to initiate each assay. A P20 pipette with the tip cut off to increase the bore was used to transfer 10 µl of worms from the top of the worm “pellet” onto the center (origin) of each rectangular plate, minimizing transfer of worm carcasses or remnant bacteria from the bottom of the tube. Typically, one tube of pre-exposed worms yielded enough for testing >100 worms per plate on 4 sets of 4 rectangular arenas (N=16). After transferring worms, 2.5 µl of the odorant in vehicle or the vehicle alone was pipetted at either end of the chemotaxis plates in opposite ends such that the worms in one set of arenas must move in the opposite direction to worms in the other set for the same response. With this orientation, if worms were responding to a gradient of an unknown cue outside the arena, then they will move in the same direction in both arenas, thereby reducing effect size in response to the odorant within the arena. The transfer of worms and odorants/vehicle typically took a total of 5 min per 4 set of 4 rectangular arenas (N=16). The plates were left with the lids on at room temperature for 1 hr. At the end of 1 hr, videos were taken of the plates keeping the extra lid underneath to allow visualization of the quadrants, and the videos were used to count the numbers of young adult worms in each quadrant. The video was taken using an iPhone positioned over the objective using an adapter and typically took 5 min per 4 set of 4 rectangular arenas (N=16).

**Statistics**

*P* -values, effect sizes, and error bars were calculated as described in Tasnim et al., 2025. The distribution of outcomes after each trial in Fig. 1 *B* was tested for significant deviations from random using the runs test (Swed and Eisenhart, 1943; Bujang and Sapri, 2018). With numbers of outcome one (n _1_ ) = 9, numbers of outcome two (n _2_ ) = 5 and numbers of runs (r) = 5, the lower critical value (L _c_ ) obtained was 3 from the table in Swed and Eisenhart, 1943. Therefore, we cannot reject the hypothesis that our series of runs can arise randomly (i.e., r = 5 > L _c_ = 3, therefore the null hypothesis for one-sided test cannot be rejected).

**Data, Materials, and Software Availability**

All data generated and the code used are available at Extended Data.

## Reagents

**Table 1. Materials and reagents**

**Table:** 

**Item**	**Catalog #**
Agar	VWR cat#97064-334 unknown lot (batch a in Fig. 1 *B* ) VWR cat#97064-334 lot#23K0156549 (batch b in Fig. 1 *B* ) Carolina® cat#842130 (batch c in Fig. 1 *B* ) VWR cat#97064-334 lot#24G2356953 (batch d in Fig. 1 *B* )
Bacto-peptone	Gibco™, Thermo Fisher Scientific cat#211677 unknown lot for Fig. 1 *B* assays 1 to 11 Gibco™, Thermo Fisher Scientific cat#211677 lot#4024890 for Fig. 1 *B* assays 12 to 14
NaCl	Supelco®, VWR cat# EMD-SX0420-1
Cholesterol	Sigma-Aldrich cat# C8667-5G
CaCl	MilliporeSigma™, FisherScientific cat#M1023820500
MgSO _4_	MilliporeSigma™, FisherScientific cat#MMX00751
K2HPO _4_	VWR Chemicals BDH® cat#BDH9266-500G
KH _2_ PO _4_	VWR Chemicals BDH® cat#BDH9268-2.5KG
Na _2_ HPO _4_ *7H _2_ O	Unknown for Fig. 1 *B* assays 1 to 8 Spectrum Chemical, The Lab Depot cat#S1400-500GM-EA for Fig. 1 *B* assays 9 to 14
KH _2_ PO _4_	VWR Chemicals BDH® cat#BDH9268-2.5KG
MgSO4	MilliporeSigma™, FisherScientific cat#MMX00751
NaOH	Supelco®, MilliporeSigma cat#SX0590-3
Ethanol	Sigma Aldrich, cat#E7023-500ML
2- butanone	Sigma Aldrich, cat#360473-500ML. Lot#SHBL7664 for Fig. 1 *B* assays 1 to 9 (age ranging from freshly opened to ~44 months at time of use) and lot#SHBQ4017 for Fig. 1 *B* assays 10 to 14 (age ranging from freshly opened to ~3 months at time of use).
35 mm plate	Falcon™ Bacteriological Petri Dishes with Lid, FisherScientific, cat#08-757-100A
100 mm plate	Falcon® Petri Dishes, VWR, cat#25373-100
Assay plate	Nunc® Rectangular Dishes, VWR cat#73521-424

## Data Availability

Description: Movie S1. Video of C. elegans washed and pooled from six 100 mm plates using M9 buffer (4x speed). Resource Type: Audiovisual. DOI: https://doi.org/10.22002/b1cfn-w5162 Description: Code used and Supplementary Dataset with raw data. Resource Type: Software. DOI: https://doi.org/10.22002/e78md-8r731
